# Operative Interference during the Second Stage of Labor

**Published:** 1894-04

**Authors:** 


OPERATIVE INTERFERENCE DURING THE SECOND STAGE OF LABOR.—McGillicuddy (*Medical Record*, October 21, 1893) states that, except in rare instances, such as cases of eclampsia, hemorrhage, or obstructions, there should be no operative interference during or before the first stage of labor. He regards the general belief that the bag of waters is of no service after the cervix has been dilated, and that the progress of labor is suspended until it is ruptured, to be erroneus. The artificial rupture of the membrane, even when the head is on the perineum, he considers not always judicious. While each case is to be treated upon its own merits, he disfavors interference so long as the child or mother is not in danger, it matters not how long the woman is in labor. In protracted labors, rather than apply the forceps, on account of the apparent suffering of the patient, and the importunities of relatives and friends, he advises the use of anodynes and anesthetics. He refers to the value of manual or bandage pressure in assisting the uterine contractions in expressing the fetus. In performing the former the hands should be spread out over the fundus, and firm but not too severe pressure made during the pains. If the patient is lying upon the side a large roller bandage or towel may be passed over the abdomen, twisted behind into a knot, and drawn tightly during each contraction. He believes three per cent. to be a fair average for interference. Mild delirium, even, he believes not to be an absolute indication for hastening the labor by instrumental interference. A rise in temperature, weakness and rapidity of the mother's pulse, or feebleness and irregularity of the infant's, he mentions as indications that there is no time for delay.

Episiotomy he regards as a doubtful operation of questionable value. In slight perineal tears, where the muscles are not deeply involved, and there is no special gaping or loss of tissue, he believes that there is no necessity for sutures. Severe or complete laceraation should always be sutured immediately, or within a few hours. He believes that version, when indicated, should always be attempted by the external manipulation. Transverse presentations are an absolute indication for external version. It is also indicated in pelvic deformity and in placenta previa, and contraindicated, as a rule, during labor.

McGillicuddy refers to the value of pelvic version, which has seldom been performed manually. Occurring spontaneously it is comparatively frequent, as Velpeau has collected one hundred and thirty-seven cases. It may be performed by external manipulation, but is preferably accomplished by a combined method. The operator should introduce his fingers in the child's groin, or over the crest of the ilium, and gradually draw the breech down into the pelvic basin. The operation of extraction can then be easily performed. If the child is large and the delivery promises to be difficult, forceps may be applied to the breech, or manual extraction employed. He recommends the selection of pelvic version, rather than podalic, in cases of transverse presentation, for the following reasons:

(1) It is a much simpler operation.

(2) It necessitates the introduction of the fingers only into the uterus, while podalic version requires the introduction of the hand, and often of most of the arm.

(3) The child need only be moved sufficiently to draw down the breech, while in most cases of podalic version the child must be made to rotate around the whole cavity of the uterus.

(4) There is less risk of rupturing the uterus.

(5) There is much less shock.

(6) The mortality to the mother and child is less.—*University Medical Magazine.*

